# The Role of Cell Surface Architecture of Lactobacilli in Host-Microbe Interactions in the Gastrointestinal Tract

**DOI:** 10.1155/2013/237921

**Published:** 2013-03-13

**Authors:** Ranjita Sengupta, Eric Altermann, Rachel C. Anderson, Warren C. McNabb, Paul J. Moughan, Nicole C. Roy

**Affiliations:** ^1^Food Nutrition & Health Team, Food & Bio-Based Products Group, Palmerston North 4442, New Zealand; ^2^Riddet Institute, Massey University, Palmerston North 4442, New Zealand; ^3^Rumen Microbiology Team, Animal Nutrition & Health Group, AgResearch Grasslands, Palmerston North 4442, New Zealand; ^4^AgResearch Grasslands, Palmerston North 4442, New Zealand

## Abstract

*Lactobacillus* species can exert health promoting effects in the gastrointestinal tract (GIT) through many mechanisms, which include pathogen inhibition, maintenance of microbial balance, immunomodulation, and enhancement of the epithelial barrier function. Different species of the genus *Lactobacillus* can evoke different responses in the host, and not all strains of the same species can be considered beneficial. Strain variations may be related to diversity of the cell surface architecture of lactobacilli and the bacteria's ability to express certain surface components or secrete specific compounds in response to the host environment. Lactobacilli are known to modify their surface structures in response to stress factors such as bile and low pH, and these adaptations may help their survival in the face of harsh environmental conditions encountered in the GIT. In recent years, multiple cell surface-associated molecules have been implicated in the adherence of lactobacilli to the GIT lining, immunomodulation, and protective effects on intestinal epithelial barrier function. Identification of the relevant bacterial ligands and their host receptors is imperative for a better understanding of the mechanisms through which lactobacilli exert their beneficial effects on human health.

## 1. Introduction

The human gastrointestinal tract (GIT) is the body's largest interface with the environment and is a dynamic barrier that harbours a complex microbial community. The intestinal epithelium allows the uptake of nutrients, secretes water and electrolytes, and simultaneously acts as a barrier to exclude pathogens and toxins [1]. Humans and their symbiotic bacteria have co-evolved and their mutual interactions are essential for human health and well-being [2]. There is increasing experimental evidence for the role played by intestinal bacteria in modulating development of the host immune system and the barrier properties of the intestinal epithelium [3].

Lactobacilli are important in the food and fermentation industries. They are also frequently used as probiotics in foods, cultured milks, and various pharmaceutical preparations [4–6]. The presence of lactobacilli is important for maintenance of the intestinal microbial ecosystem and for providing protection against pathogen infection [7–9]. Lactobacilli are present throughout the GIT in varying proportions. They are dominant in the proximal small intestine [10], a nutrient rich environment, whereas in the faecal microbiota they are present at most ~0.01%–0.6% and this proportion varies significantly between individuals [11, 12]. They have the ability to adhere and interact with the epithelium and the mucosal layers, while surviving the hostile conditions of the luminal environment and the competing microbiota [13]. These properties add to their potential to be used as probiotics that fit the parameters set by the Operating Standards in 2002 (FAO/WHO: Guidelines for the evaluation of probiotics in food). However, studies have shown that different strains of lactobacilli can evoke different responses in the host and therefore, the results from one strain cannot be generalised to others [9]. 

Adherence of lactobacilli to the intestinal epithelium is an important characteristic as it promotes persistence time and colonisation, stimulates microbe-host interactions through immunomodulation, and provides protection to the intestinal barrier by various mechanisms including antagonistic activities against pathogens [14]. Bacterial cell surface components (adhesins, polysaccharides, and proteins) play major roles in the adherence of lactobacilli to the intestinal epithelium, interactions that might lead to pathogen exclusion and immunomodulation of host cells [15, 16]. The adhesive properties of lactobacilli are directly linked to their surface properties which are influenced by the structure and composition of their cell wall. Several studies implicate cell surface components, either individually or collectively, in microbe-host interactions [17, 18].

Lactobacilli show great diversity in cell surface architecture and are known to modify their surface properties in response to environmental changes [19, 20]. Different macromolecules constituting the cell wall of lactobacilli have been shown to contribute to maintaining bacterial cell integrity during environmental stress [21]. The cell surface architecture of lactobacilli and their ability to express certain surface components, or to secrete specific compounds that act directly on the host cells, may thus influence the physicochemical properties of the bacterial cell and strain-specific properties.

This paper will focus on cell surface components of lactobacilli that influence host response and impart strain-specific characteristics to lactobacilli.

## 2. Cell Surface Structures

**Figure 1:**
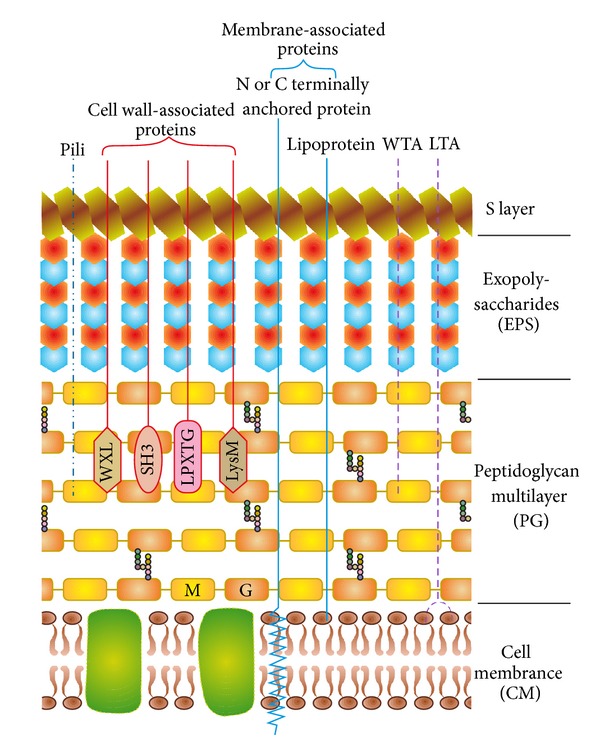
Cell envelope of lactobacilli with a schematic representation of cell-wall and membrane-associated proteins (the figure was adapted from [35, 153]). The bilipidic cell membrane (CM) with embedded proteins is covered by a multilayered peptidoglycan (PG) shell decorated with lipoteichoic acids (LTA), wall teichoic acids (WTA), pili, proteins, and lipoproteins. Exopolysaccharides (EPS) form a thick covering closely associated with PG and are surrounded by an outer envelope of S-layer proteins. The proteins are attached to the cell wall either covalently (LPXTG proteins) or noncovalently (exhibiting LysM, SH3, or WXL domains), lipid anchored to the CM (lipoproteins) or attached to the CM via N- or C-terminal transmembrane helix. M: N-acetyl-muramic acid; G: N-acetyl-glucosamine.

The cell envelope of lactobacilli, like that of all lactic acid bacteria, is composed of the bilipidic plasma membrane with embedded proteins encompassed by the cell wall. The bacterial cell wall consists of a thick multilayered sacculus made of peptidoglycan (PG), decorated with teichoic acids (wall teichoic acids (WTA) and/or lipoteichoic acids (LTA)), exopolysaccharides (EPS), proteinaceous filaments called pili, and proteins that are anchored to the cell wall through different mechanisms (Figure 1). Some species of lactobacilli display an additional paracrystalline layer of proteins surrounding the PG layer, referred to as the S-layer. These macromolecules together may play crucial roles in determining species and strain-specific characteristics of lactobacilli by influencing host-microbe interactions and microbial adaptations to the changing host environment.

### 2.1. Peptidoglycan

PG is the largest component of the bacterial cell wall and is an essential polymer in lactobacilli that determines the shape and preserves the integrity of the bacterial cell. The PG layer has been described as a fisherman's net, functioning both as a container for and a sieve to the bacteria [24]. The elastic nature of PG helps withstand stretching forces caused by bacterial turgor pressure, excludes large molecules from entering the bacterial cell, and at the same time restricts secretion of large proteins. Proteins with theoretical molecular mass as large as 49.4 KDa and 82.1 KDa have been reported to be secreted by *Lactobacillus rhamnosus* GG and *Lactobacillus plantarum,* respectively [25, 26]. Some large proteins are unable to diffuse through the cell wall and are dependent on the cell wall expansion process to be dragged to the outer surface of the thick PG layer before being passively released into the external milieu [24, 27]. The threads of this net are polymers of covalently linked alternating residues of N-acetyl-glucosamine (GlcNAc) and *β*-1-4-linked N-acetyl-muramic acid (MurNAc). The glycan strands are held together by crosslinking pentapeptide side chains providing elasticity to the net. The pentapeptide side chain is made of alternating L- and D-amino acids and this attaches to the D-lactyl carboxyl group of MurNAc. Considerable variations occur in the basic compositions of the glycan strands and pentapeptides which impart strain-specific characteristics to the bacteria [28, 29]. Following biosynthesis, assembly, and incorporation of the PG subunits, modifications in the GlcNAc and MurNAc structures can occur and affect interactions between host and lactobacilli [24]. These modifications include removal of acetyl groups from cell wall PG [30], 6-O-Acetylation of cell wall MurNAc residues [31], and the substitution of C6 of MurNAc by teichoic and teichuronic acids [32]. These modifications can affect the physiology of the bacterial cell wall by increased sensitivity to autolysis, resistance to lysozyme, and hydrophobicity of the cell envelope which in turn affects recognition by host receptors and bacterial adhesion [33, 34]. 

### 2.2. Teichoic Acids

Teichoic acids (TAs) are the second major component of cell walls of lactobacilli and account for up to half of the cell wall dry weight [35]. They are anionic polymers made of repeating units of glycerol- or ribitol-phosphate, covalently linked to PG as WTA or attached to the cytoplasmic membrane through their lipid anchors as LTA [36–38]. A fraction of LTA can be found free in the cell wall or may be released into the extracellular medium through deacetylation of the lipid anchor, where they are recognised as ligands by receptors present on intestinal epithelial cells [3]. LTAs contribute to the anionic character of the cell wall and provide hydrophobicity, which in turn influences the adhesiveness of the cell wall [34].

The overall structure of TA is a chain made of phosphodiester-bound glycerol or ribitol residues hooked through a terminal “linkage unit” on the C6 of the MurNAc residue of a growing PG chain. The structure of the linkage unit is well conserved and is made of a disaccharide N-acetylmannosaminyl *β* (1–4) glucosamine followed by glycerol phosphate. The variety of TA can occur in the nature of the sugars and number of phosphate residues. There are considerable variations in structure and abundance between WTA and LTA molecules. Their size and physicochemical properties depend on several factors such as species or strain, stage or rate of growth, availability of phosphate, acidity of medium, and carbon source, and so forth [24]. Although all lactobacilli have TA in their cell walls, not all *Lactobacillus* cell walls contain WTA and some species appear to contain only LTA [39]. TA can function as a reservoir for phosphates and also as a scavenger of cations (Mg^++^ in particular) [40, 41]. TAs can also help in creating a pH gradient across the cell wall and are also known to be involved in phage adsorption and autolysin activity [42]. Glycosylated TAs have been reported to be essential for the adsorption of some *L. plantarum *phages, and studies with *L. delbrueckii* subsp. *lactis* show the involvement of LTA in phage inactivation [43].

### 2.3. Cell Wall Polysaccharides

Cell wall polysaccharides are neutral polysaccharides that can either form a thick outer capsule closely associated with the cell wall and often be covalently bound to MurNAc of PG (referred to as capsular polysaccharide; CPS) or be loosely associated with it (wall polysaccharides; WPS) or be released into the extracellular medium (EPS) [44, 45]. Distinction between these various classes of cell wall polysaccharides is often difficult. In lactobacilli, EPS usually refers to extracellular polysaccharides that can be attached to the cell wall or released into the surrounding medium. The complex variations in the composition of EPS, which differs in the nature of the sugar monomers along with their linkages, distribution, and substitution, add to the structural variety of the *Lactobacillus* cell wall [46, 47]. EPS is generally composed of heteropolysaccharides consisting of different sugar moieties such as glucose, galactose, rhamnose, GlcNAc, and N-acetylgalactosamine [48]. Residues of glucuronic acid, phosphate, acetyl, and pyruvate groups may also be present in some strains of lactobacilli. In addition to the heteropolymeric EPS molecules, some strains of lactobacilli are capable of synthesising homopolysaccharides such as glucans or fructans from sucrose [49].

Studies with *L. rhamnosus* GG identified two different classes of EPS: long galactose rich molecules and short glucose/mannose rich EPS molecules [50]. Some polysaccharide chains can also be present as glycoproteins, providing anchorage to S-layer proteins, creating an extra level to the complexity of the bacterial cell wall architecture [50]. Specific contributions of EPS to cell wall functionality are unclear, although their general role is to mediate interactions of lactobacilli with environmental components and promote bacterial adhesion and biofilm formation to inert or living surfaces [51, 52].

### 2.4. Pili and Flagella

Pili are multisubunit protein polymeric structures that have been functionally analysed and characterised only in *L*. *rhamnosus* GG [53, 54], although they have been identified at the genome level in some lactobacilli [23]. These nonflagellar appendages are an assembly of multiple pilin subunits that are covalently coupled to each other by the transpeptidase activity of the pilin-specific sortase [53, 55]. The resulting isopeptide bonds are formed between the threonine of an LPXTG-like motif and the lysine of YPKN pilin motif in the pilin subunits [56]. After assembly, the pilins are attached to the cell wall by a membrane bound transpeptidase, the housekeeping sortase [57]. The roles of pili in bacterial adhesion, invasion, aggregation, formation of biofilms, and modulation of immunity are well established [58, 59] but the receptors in the host that recognise these pili are still unknown and their function in signalling host response is unclear. The presence of flagella is an unusual feature found in lactobacilli and at present, at least twelve motile species of lactobacilli have been recognised [11]. The bacterial flagellum comprises of polymers of protein called flagellin, which is suggested to act as a ligand and mediate activation of signalling pathways and modulation of host immune cells [60].

### 2.5. Cell Surface Proteins

The cell surface proteins in lactobacilli are either anchored to the cell wall by various mechanisms or secreted from the bacterial cell into the surrounding medium, where they reassociate with the cell wall through electrostatic interactions [61]. Cell surface proteins include the S-layer proteins which constitute the major cellular proteins that surround the cell. Examples of cell surface proteins include the 43 KDa collagen binding S-layer protein from *L. crispatus* and cell surface proteins of 15 kDa and two proteins of 45 and 58 KDa from *L. acidophilus *CRL639 that are involved in binding to fibronectin and collagen [62]. Covalently anchored proteins are further subcategorised into N- or C-terminally anchored proteins, lipid-anchored proteins (lipoproteins), and LPXTG-anchored proteins. The N-terminally anchored proteins represent the largest group of cell-surface-anchored proteins in lactobacilli and are mainly involved in cell-envelope metabolism, extracellular transport and signal transduction, competence, and protein turnover [35, 63]. Many C-terminally anchored proteins, linked to the cell membrane through C-terminal transmembrane domains, are encoded by lactobacilli, but the function of several of these proteins remains unclear [35]. The lipid-anchored proteins constitute the second largest group of predicted membrane-anchored proteins in lactobacilli, and are involved in transport, adhesion, antibiotic resistance, sensory processes, homeostasis of the cell envelope and secretion, folding and translocation of proteins [35, 64]. The C-region of the signal peptide of these lipoproteins contains the lipobox motif [L-(A/S)-(A/G)-C]. Lipidation followed by cleavage at the N-terminal of the Cys-residue in the lipobox results in the covalent binding of the lipoprotein to the cell membrane through a thioether linkage [65]. LPXTG-anchored proteins or sortase-dependent proteins (SDP) are covalently attached to the PG and reportedly play a crucial role in lactobacilli-host interactions [66]. These proteins typically contain a cleavage site, an LPXTG motif, located in the C-terminal region of the mature domain, followed by a stretch of hydrophobic residues and a positively charged tail [66]. The LPXTG motif is recognised by the sortase (SrtA) enzyme, which cleaves between the T and G residues and then covalently links the threonine carboxyl group to an amino group of the PG cross-bridges [67]. Although SrtA recognises the sequence LPXTG, another sortase, called SrtB in *S. aureus,* has been reported to recognise and process proteins bearing the sequence NPQTN [68]. Recent studies involving cross-linked protein products of SrtA and SrtB indicate that different types of sortases may be able to attach proteins to distinct positions within the cell wall [69].

Noncovalently anchored proteins are bound to the bacterial cell surface through binding domains. Some proteins can also be found anchored to other cell wall proteins through protein-protein interactions, while others are known to reassociate with the cell wall after being secreted, through electrostatic interactions [70].

Many species of lactobacilli display a surface coating made of a crystalline, two-dimensional array of protein or glycoprotein subunits assembled in lattices with different symmetries, also referred to as the S-layer. Lactobacilli S-layer proteins represent up to 10 to 15% of total cell wall proteins. These proteins are highly basic, with stable tertiary structures ranging from 40 to 60 kDa [3]. S-layer proteins are the most prominent glycoproteins in prokaryotes, and although in lactobacilli most S-layer proteins appear to be nonglycosylated, some lactobacilli have glycosylated S-layer proteins that have been identified [71]. S-layer proteins are relevant to cell wall polysaccharide pyruvylation and are noncovalently bound to the underlying PG cell wall, generally through secondary polymers such as LTA, WTA, and neutral polysaccharides [70]. Properties such as adhesion, aggregation, and pathogen inhibition have been related with the occurrence of particular types of S-layers, although S-layer functions in lactobacilli are not just species but also strain specific. Studies indicate that there is a correlation between the different structural and chemical characteristics of the S-layer proteins with the surface properties of lactobacilli [50, 70]. There is ample evidence of S-layer proteins influencing the development of microbial communities as biofilms and therefore, it is likely that S-layer proteins have a role in the interaction of lactobacilli with other microorganisms [72].

Lactobacilli have enzymes with binding domains that help to keep them anchored to the bacterial cell surface. For example, extracellular enzymes such as autolysins display a stretch of 20 amino acids that have conserved multiple tandem repeats of aromatic residues and glycines that anchor to the bacterial cell surface by binding to the choline residues of WTA and LTA [73]. The LysM domain (lysine motif) is found in many extracellular enzymes that are suggested to have a PG binding function and are involved in cell wall metabolism [74]. WXL domain-containing proteins were identified in lactobacilli based on *in silico* analysis [75] and are suggested to interact with the PG layer through their protein C terminus. This domain has also been reported to mediate noncovalent binding between the bacterial cell wall of *Enterococcus faecalis* and other Gram-positive bacteria [76]. SH3b domains have been identified in some lactobacilli and are proposed to be involved in cell wall turnover. They have been suggested to recognise specific sequences within the peptide cross-bridges of the PG, thus targeting and binding to the cell wall [77]. A putative domain composed of three *α*-helices at the C- or N-terminal of an extracellular protein has been reported in some lactobacilli (*L. plantarum, L. johnsonii, L. casei, L. brevis, L. helveticus, *and* L. gasseri*) and is suggested to be involved in cell wall degradation through binding to the PG [35].

## 3. Cell Surface Adaptations of Lactobacilli in Response to the Host Environment

The cell envelope is the first target of physicochemical and environmental stress. Lactobacilli encounter several environmental stress factors during their transit through the GIT such as low pH, bile salts, and oxidative and osmotic stress, along with starvation stress. Lactobacilli have developed sophisticated responses and adaptations to survive these stressors. Stress responses of lactobacilli rely on the coordinated expression or suppression of genes that act in concert to improve stress tolerance. These genes can alter cellular processes such as cell division, membrane composition, transport systems, housekeeping, and DNA metabolism and are regulated by factors that can control several genes and sometimes even other regulators. Lactobacilli respond to stress in specific ways dependent on the strain, species, and the type of stress. The coordination of these stress responses is achieved by the network of regulators that allow the bacterial cell to react and adapt to different stressors. 

### 3.1. Acid and Bile Stress

Survival under acidic conditions is achieved by adapting to low pH through a mechanism called acid tolerance response (ATR). Studies with acid- and bile-resistant variants of *L. acidophilus* suggest that an inducible pre-existing system co-exists with a *de novo* protein synthesis mechanism, which together protect against acid stress [78]. Bile acids are conjugated to glycine or taurine in the liver and enter the intestine where the amino acid may be hydrolysed by bile salt hydrolases (BSH) expressed by bacteria, including lactobacilli. In *L. plantarum*, the capacity to tolerate taurodeoxycholic acid (TDCA) has been attributed to the expression of TDCA hydrolase, but other studies have shown that BSH activity and resistance to bile are unrelated properties in lactobacilli [79, 80]. Many resistance mechanisms resulting in alteration of lactobacilli cell surface structures are common for bile and acid stress [81]. The macromolecules composing the bacterial cell envelope (cell wall and cell membrane) contribute to maintaining the cell integrity under these stress situations. For instance, bile salts and cholesterol have been shown to induce changes in the lipid cell membrane of *L. reuteri *[19] while low pH causes alterations in the fatty acid composition of an oral strain of *L. casei *[20]. 

 Screenings of acid responses and bile salt responses in lactobacilli have identified genes involved in PG biosynthesis and cell envelope functions. Gene expression analysis of *L. acidophilus* identified a high number of genes involved in PG and cell surface protein (e.g., SrtA) biosynthesis that are differentially expressed after bile exposure [82]. In *L. reuteri*, the response to acidic conditions involves the ClpL chaperone, an ATPase with chaperone activity and a putative cell wall-altering esterase. These enzymes are also reported to be induced by bile exposure, further implying common resistance mechanisms for acid and bile stress [83, 84]. Other cell surface structures (LTA, WTA, and EPS) have also been suggested to play roles in proper functioning of cell integrity in acidic conditions and in the presence of bile [85]. EPS biosynthesis also reportedly involves suppression of genes after bile exposure as noted in *L. acidophilus* and *L. reuteri*, although the role of EPS in bile and acid resistance is still unclear [82, 84]. 

### 3.2. Oxidative and Osmotic Stress

In addition to acid and bile stress, the survival capacity of lactobacilli to oxidative and osmotic stress in the GIT is important. Oxidative stress that can adversely affect cell fitness is caused by exposure to reactive oxygen species (ROS) resulting from partial oxygen reduction to superoxide anion radicals (O_2_), hydroxyl radicals (^*∙*^OH), and hydrogen peroxide (H_2_O_2_). Polyunsaturated fatty acids are sensitive to ROS attack and the resulting peroxidation of membrane lipids and protein alteration affect cell membrane permeability and osmoregulation [86]. To minimise the damage caused by ROS, lactobacilli counteract ROS generation with the help of enzymes such as catalase, NADH oxidase/peroxidise, and superoxide dismutase (SOD) or nonenzymatic compounds such as ascorbate, glutathione, and Mn^2+^. Resistance to oxidative stress varies widely between species and strains. Stress handling mechanisms range from preventing formation of ROS, elimination of ROS, and defence against oxidative damage to repair of oxidative damage [87].

The fatty acid composition of the cell membrane of *L. helveticus* has been shown to change underoxidative stress and this was reported to be due to an increased activity of the O_2_-consuming fatty acid desaturase system which reduces the free radical damage in the cell [21]. Interestingly, bile stress has also been shown to induce oxidative stress, and studies indicate that the expression of glutathione reductase is influenced by bile treatment [88].

Lactobacilli are often exposed to changes in the osmolarity of their environment which can compromise essential cell functions. Changes in solute concentrations in the environment cause changes in cell turgor pressure which lead to changes in cell volume. To maintain turgor pressure and retain water in the cell, lactobacilli accumulate compatible solutes under hyper-osmotic conditions and release them under hypo-osmotic conditions. In *L. acidophilus*, disruption of the cell division enzyme CdpA caused an increased resistance to bile salts while showing reduced resistance to osmotic stress. Similar effects were shown by the SlpA mutant of *L. acidophilus*, which was more sensitive to osmotic stress while being more resistant to bile. According to these studies, certain components of the cell wall remain uncleaved or cross-linked resulting in an immature structure of the cell wall in the mutant thus altering its phenotype [89, 90]. Studies with *L. alimentarius *showed that when grown under sublethal doses of NaCl, an increased tolerance was observed towards hyper-osmotic conditions or an increased ATR against organic acids. Similar cross-protection was observed when the cells were exposed to sublethal doses of these acids implying that common mechanisms were involved [91].

### 3.3. Starvation Stress

The capacity to adapt to a specific nutritional environment is important to lactobacilli and ensures their residence time and survival in the GIT. Starvation is one of the most common stresses faced by lactobacilli and bacterial growth leading to nutrient exhaustion, accumulation of fermentation end product (e.g., lactic acid), and subsequent starvation contributes to this stress. Nutrient starvation in lactobacilli has been mainly studied by limiting the supply of carbohydrate, phosphate, and nitrogen. Lactobacilli adapt to these nutritional limitations by either downregulating nucleic acid and protein synthesis and/or protein degradation and amino acid synthesis [92]. Moreover, extreme environmental stress conditions can indirectly provoke starvation by decreasing the activity of transporters resulting in reduced availability of essential nutrients that might be present in the extracellular environment [93]. Nutrient starvation leads to growth arrest, and different lactobacilli have developed different strategies to survive starvation. Modification of cell morphology and cell division at the entry of the stationary phase, resulting in diminished cell size, has been reported in lactobacilli under these conditions [87].

Starvation resistance mechanisms in lactobacilli are diverse as they occupy different niches and do not encounter the same starvation conditions. It is well established that bacteria become more resistant to various types of stresses and develop a general stress-resistant state on entering the stationary phase. Carbohydrate starvation induces increased resistance to many stress conditions. Amino acid catabolism, in particular arginine degradation, plays a role in the enhanced survival of *L. sakei* during stationary phase [94]. In *L. acidophilus*, 16 proteins were reported to be synthesised as a response to starvation, of which 7 were induced by stationary phase while the others in response to low pH [95]. In *L. lactis*, glucose starvation was shown to induce resistance to many stresses (heat, low pH, and oxidative and osmotic stress) [96]. Similarly in *L. bulgaricus*, lactose starvation increased resistance to heat, acid, and bile stress [97]. The regulation of starvation-induced proteins in lactobacilli is still unclear. Although studies indicate a small overlap between stress-specific and starvation regulator genes and many proteins can be commonly induced by more than one stress, only a few proteins are common to all stresses.

## 4. Lactobacilli and Host Interactions Involving Bacterial Cell Surface Factors

The human GIT represents the first line of defence against bacteria, viruses, fungi, and parasites that can act as pathogens. The GIT epithelium is also associated with indigenous commensal microorganisms that comprise the microbiota. Thus, the epithelium is important for the maintenance of GIT homeostasis in the presence of commensal microorganisms while preventing pathogen invasion [98]. Lactobacilli interact with the intestinal epithelium through several mechanisms that help modulate the immune response of the host, preserve barrier integrity, and maintain microbial balance through exclusion of pathogens by direct antimicrobial activity (production of bacteriocins or inhibitors), competitive exclusion (competing for binding sites), and/or stimulating anti-inflammatory immune responses (Table 1).

### 4.1. Adherence

Adherence of bacteria to the GIT mucosa is an important factor for colonisation and leads to direct interactions that can result in competitive exclusion of pathogens and the modulation of host response. Adhesive mechanisms of human pathogenic bacteria have been studied extensively through the use of *in vitro* model systems. Human colorectal adenocarcinoma cell lines such as Caco-2 or HT-29 cells, immobilised intestinal mucus and extracellular matrices, quantitative measurements, microscopic enumeration, and immunological detection methods have been used for assessing adhesive mechanisms [120, 121]. However, knowledge of the bacterial cell surface molecules mediating adhesion to the GIT mucosa is still limited. Genomics-based approaches have revealed several bacterial cell-surface-associated proteins that bind to mucus and intestinal cells [17]. Lactobacilli adhesins have been grouped into mucus binding proteins; sortase-dependent proteins; S-layer proteins; proteins mediating adhesion to extracellular matrix (ECM) components of the intestinal epithelial cells; nonprotein adhesins (LTA and EPS).

Intestinal epithelial cells form a barrier between the host and the content of the lumen and are covered by a protective layer of mucus. The mucus layer exists in a dynamic equilibrium, balanced between production, degradation, and physical erosion. It provides bacteria with only a short residence time in the GIT upon adhesion, thereby protecting the host against pathogens and undesirable bacterial colonisation [122]. However, the mucus layer also provides a habitat for commensal bacteria, such as lactobacilli. Adherence of lactobacilli to mucus has been experimentally validated *in vitro* using adhesion assays with probiotic-pretreated intestinal mucus glycoproteins [123], as well as *in vivo* by microscopic analysis of biopsy samples [124]. *Lactobacillus* adhesion to mucus involves mucus binding proteins (Mubs) which in addition to the same domain organisation typical of cell surface proteins (the N- terminal signal peptide and C terminal LPXTG anchoring motif) share a mucus binding domain. Mubs are encoded by Lactobacillales-specific clusters of orthologous protein coding genes (LaCOG) and contain one or more Mub repeats. Proteins containing Mub repeats are abundant in lactobacilli that inhabit the GIT, suggesting that Mub repeat is a functional unit that may be an evolutionary adaptation for survival in the GIT. A database search using the sequence from the extracellular Mub domain of *L. reuteri *[125] and *L. acidophilus *[17], and the lectin-like mannose-specific adhesin (Msa) of *L. plantarum *[103], resulted in the identification of proteins containing multiple Mub domains in several species of lactic acid bacteria (LAB), further suggesting that this domain is a LAB-specific functional unit. Studies with *L. fermentum* BCS87 have helped identify and characterise a 32 KDa surface-associated protein (32-mMubp) that is suggested to mediate adhesion to mucus [114]. The Mub domain consists of a series of amino acid residues, varying in size from 100 to 200 residues per domain [126]. Studies have shown that Mub and Mub-like proteins contribute to mucus binding and autoaggregation, but high genetic heterogeneity among strains results in strain-specific diversity in adhesion to mucus [122].

Some lactobacilli (e.g., *L. rhamnosus* GG) have fimbriae (also called pili) that reportedly enhance adhesion to mucus glycoproteins of the host cells with subsequent colonisation of the GIT [53]. Studies with *L. rhamnosus* GG have shown a mucus binding factor (MBF) with a presumed ancillary involvement in pilus-mediated mucosal adhesion [107]. However, fimbriae of some Gram-positive pathogens were shown to induce pro-inflammatory responses [127], while capsular polysaccharide of *L. rhamnosus* GG was found to shield fimbriae, possibly suppressing pro-inflammatory responses [99]. Such role and possible positive effects of *L. rhamnosus* GG fimbriae are still unclear and need to be validated.

In lactobacilli, a subgroup of surface proteins that contains the LPXTG motif at their C terminal is recognised by SrtA. SrtA cleaves those proteins and anchors the resulting product to PG, thus incorporating these SrtA-dependent proteins on the microbial surface. Although many sortase-dependent proteins are encoded by lactobacilli, the majority have no assigned function. Of the functionally characterised proteins belonging to this family, three correspond to the mucus adhesins of *L. reuteri* (Mub), *L. plantarum* (Msa), and *L. acidophilus* (Mub). LspA, of *L. salivarius *UCC118, is the fourth characterised sortase-dependent protein that also binds mucus and is known to mediate adhesion of this species to intestinal epithelial cells [109, 128]. Recent studies with *L. casei *BL23 sortases and SrtA mutants suggest that SrtA might be involved in adhesion of this strain to Caco-2 and HT29 cells [117]. Although most sortase-dependent proteins of lactobacilli seem to have mucus-binding capacity, not all of them have affinity to mucus components and the function of putative lactobacilli sortase-dependent proteins remains unclear [16]. Domain analysis and phylogenetic profiling of the extracellular proteins of *L. plantarum* involved in adhesion reported 10 of the 12 identified proteins to contain the LPXTG motif. Their predicted role was adherence to collagen, fibronectin, chitin, or mucus [75]. Of these 12 identified proteins, the role of Msa from *L. plantarum *in adhesion has been experimentally validated, but the roles of the other *in silico* identified putative adhesins are speculative and need *in vitro* and *in vivo* validation.

S-layer proteins form the outermost interacting surface in different species of lactobacilli and have been shown to act as adhesins to epithelial cells and components like mucus and extracellular matrix proteins. The role in adhesion of S-layer proteins of *L. acidophilus* (SlpA), *L. crispatus *(CbsA), and *L. brevis* (SlpA) has been experimentally validated [63, 110, 129]. The removal of the S-layer that reduced bacterial aggregation in *L. acidophilus, L. kefir,* and *L. crispatus* suggests their functional involvement in this process [112, 113]. There is considerable evidence that aggregation directly influences the development of structured microbial communities as biofilms, and the removal of the S-layer completely abolishes coaggregation, thus suggesting that it is mediated by S-layer proteins. Studies also suggest that S-layer proteins with lectin-like activity interact with glycoproteins and polysaccharides and thus influence interactions of lactobacilli with other microorganisms [113].

Aggregation helps to form a physical barrier thus preventing colonisation by pathogens. Immunoblotting assays show direct interaction between *L. kefir* S-layer proteins and *Salmonella* surface adhesins. Pretreatment of *Salmonella* with purified S-layer proteins has been shown to protect two human intestinal epithelial cell lines, parental Caco-2 and the TC-7 clone, from *Salmonella* invasion, but the protective effect was not observed when *Salmonella* was pretreated with nonaggregative strains [112]. These observations strengthen the theory that coaggregation prevents invasion by *Salmonella* and protects epithelial cell damage. In *L. kefir*, the S-layer also influenced hemagglutinating, but not adhesion to Caco-2 cells, unlike the S-layer of some strains of *L. acidophilus* that are involved in both Caco-2 adhesion and aggregation [100, 112, 113]. In *L. crispatus*, the removal of the S-layer did not affect autoaggregation or hemagglutinating [63], suggesting that the S-layer may not be the only structure involved in these processes and that other covalently bound proteins or molecules such as LTA or lectin-like molecules can mediate adhesion to intestinal epithelial cells.

The extracellular matrix (ECM) is a complex structure surrounding intestinal epithelial cells and is composed of various proteins such as laminin, fibronectin, and collagen. Some lactobacilli can bind to these proteins, thus competing with pathogens that have ligands for the same binding sites [62]. Examples of ECM binding adhesins are the fibronectin-binding protein (FbpA) of *L. acidophilus* and the collagen-binding protein (CnBP) of *L. reuteri *[17, 130]. Pfam domain analysis of CnBP predicted a bacterial extracellular solute-binding domain (PF00497) that was also detected in mucus adhesion promoting protein (MapA), which was found to be a homologue for CnBP. Although MapA reportedly mediates the binding of *L. reuteri* to Caco-2 cells and mucus, database analysis detected no mucus binding proteins, suggesting a role for the extracellular solute-binding domain of MapA in adhesion [16]. Other examples include the previously discussed S-layer proteins.

*Lactobacillus* adhesion to the GIT has also been shown to involve surface-associated nonprotein factors such as the LTAs and EPS. LTAs contribute to the anionic character of the cell wall and provide hydrophobicity, which in turn influences the adhesiveness of the cell envelope [34]. EPS may contribute to the physicochemical properties of the cell surface by shielding other cell surface adhesins, acting as ligands mediating adhesion and coaggregation [131, 132]. In *L. acidophilu*s BG2FO4, carbohydrates on the bacterial cell wall were reported to be partly responsible for adhesion of this strain to Caco-2 cells and to mucus secreted by the mucus producing human adenocarcinoma cell line HT29-MTX cells [101]. In *L. johnsonii*, LTA has been reported to mediate adhesion to Caco-2 cells [18] and in *L. acidophilus*, different types of exopolysaccharides have been shown to influence adhesion to ECM components [62]. 

Two peculiar examples of cytoplasmic-localised proteins that act as surface-translocated adhesins in lactobacilli are elongation factor Tu (EF-Tu) and the heat shock protein GroEL of *L. johnsonii*. EF-Tu is involved in protein biosynthesis in the cytoplasm but has been reported as surface translocated in many lactobacilli. In *L. johnsonii*, surface translocated EF-Tu fulfills an alternative role of mediating adhesion to intestinal epithelial cells and mucins. GroEL is a mediator of protein folding but when localised at the bacterial surface, it mediates adhesion to human intestinal cells and mucins [115, 116]. No domains or motifs have been found in either protein to account for their translocation across membranes. A cell-surface-associated enzyme GAPDH of *L. plantarum* LA318 has been found to mediate adherence to human colonic cells supposedly by recognising the sugar chains on the mucus and acting as a lectin-like protein [104]. GAPDH is surface localised although it lacks the conventional N-terminal signal sequence or a membrane anchoring motif.

### 4.2. Maintenance of Epithelial Barrier Function

There is increasing evidence that lactobacilli may have beneficial influences on the intestinal epithelium. The role of lactobacilli in maintaining the intestinal barrier function is achieved by various mechanisms such as inducing mucus production, modulation of cytoskeletal, and tight junction protein phosphorylation, which can enhance tight junction function, immune response, and preventing apoptosis of the intestinal epithelial cells. Enhancement of epithelial barrier integrity by lactobacilli has been observed in both *in vitro* and *in vivo* models. For example, *L. brevis* strengthens epithelial barrier function in healthy rats as assessed by mannitol permeability, with mannitol being used as a probe to study colonic wall permeability [111]. Administration of *L. plantarum* and *L. reuteri* to rats with methotrexate-induced enterocolitis improves bowel barrier function [105]. *L. plantarum* has also been shown to increase epithelial barrier integrity using transepithelial electrical resistance assays as a measure of the integrity of tight junctions between intestinal epithelial cells with Caco-2 cell line as a model [106]. Studies with interleukin-10 gene-deficient (IL-10^−/−^) mice indicate that most of them develop chronic enterocolitis, as IL-10 has been suggested as an essential immunoregulator in the GIT and is a potent suppressor of macrophage and T-cell functions. *Lactobacillus* species have been shown to prevent chronic colitis in IL-10^−/−^ mice [133]. Studies with human intestinal epithelial HT29 cells show that the lipid moiety of LTA from *L. johnsonii* and *L. acidophilus* inhibits *E.coli* and lipopolysaccharide- (LPS-) induced IL-8 production (IL-8 is a chemokine and is a potent promoter of angiogenesis) by epithelial cells thus identifying important bacterial cell surface factors that confer beneficial effects on the GIT [102]. Recent studies with *L. rhamnosus* GG using Caco-2 epithelial cells validate that the lipid chains of LTA are needed for IL-8 mRNA expression and that D-alanine substituents are also important for IL-8 induction in Caco-2 cells [134].

The intestinal epithelial barrier is also affected by alterations in mucus and chloride secretion by epithelial cells. Mucin forms a physicochemical protective barrier for the underlying intestinal epithelial cells and assists in the prevention of mechanical, chemical, enzymatic, and microbial damage to the intestinal barrier and also restricts microbial invasion following adherence [135]. *In vitro* experiments with selected *Lactobacillus* strains have shown that adherence of enteropathogenic *E. coli *to human intestinal epithelial cells is inhibited by induction of intestinal mucin gene expression [136]. Mucin is known to inhibit bacterial translocation, and studies with *L. casei* LGG showed increased expression levels of mucin genes in a Caco-2 cell model [118]. Expression of mucin genes, induced by lactobacilli, has been shown to be dependent on direct cell contact between *L. plantarum *and intestinal epithelial cells [136].

In addition to mucus production, modulation of tight junction protein expression in epithelial cells is an important factor in preserving epithelial barrier integrity. Tight junction proteins are dynamic structures that bind together epithelial cells at their apical junctions and help maintain barrier integrity. Structural changes in tight junction proteins influence their functionality. Zonula occludens-1 (ZO-1), a tight junction protein, and F-actin, a structural component of the epithelial cell cytoskeleton, are known to play important roles in maintaining cytoskeleton architecture of epithelial cells thus preserving barrier integrity. *L. acidophilus* has been shown to prevent disruption of the distribution of ZO-1 and occludin by *E. coli* and enhance cytoskeletal and tight junction protein structures such as occludin and actinin in intestinal epithelial cells [137]. Lactobacilli also improved barrier function in rats by increasing occludin expression and maintaining epithelial tight junctions [138, 139].

The adherence ability of lactobacilli enables them to compete with pathogenic bacteria for receptors that are expressed on intestinal epithelial cells, thus shielding them from damage caused by pathogenic bacteria and preserving barrier integrity [22, 140]. *L. rhamnosus* R0011 and *L. acidophilus *R0052 inhibit infection of intestinal cells caused by exposure to *E. coli* by reducing bacterial adhesion and cytoskeletal rearrangements [140]. Studies with specific lactobacilli strains show that direct cell contact is needed to induce expression of opioid and cannabinoid receptors in intestinal epithelium mediating analgesic functions in the GIT implying involvement of cell-surface-related effector molecules [141]. Antiapoptotic effect of* L. rhamnosus* GG in intestinal epithelial cells is also dependent on direct cell contact [102]. The activation of the antiapoptotic Akt/protein kinase B and inhibition of the activation of proapoptotic p38/mitogen-activated protein kinase by cytokines were suggested to prevent apoptosis in the intestinal epithelial cells [108].

### 4.3. Immunomodulation

Lactobacilli are able to modulate immune responses of the host by interaction with the GIT mucosa. Bacterial surfaces exhibit characteristic features known as microbe-associated molecular patterns (MAMP), which are usually cell wall components, such as LPS, PG, LTA, and WTA, but can also be lipids, lipoproteins, proteins, and nucleic acids [142, 143]. MAMPs are recognised by various pattern recognition receptors (PRR) that are expressed by many cell types including immune cells, intestinal epithelial cells, and nonimmune cells. Recognition of these MAMPs by PRRs induces a signalling cascade that can result in the production of cytokines, chemokines, and other effector molecules thus activating the innate immune response in the host. PRRs include toll-like receptors (TLR), nucleotide oligomerization domain (NOD)-like receptors (NLR), and C-type lectin receptors (CLR). Of these, TLRs and NLRs are well-characterised receptors of the host immune system that are known to interact with bacterial cell surface components like the LTA and PG [64]. TLR signalling pathways involve the recruitment of adaptors such as myeloid differentiation primary response gene 88 (MyD88), which in turn activates the mitogen-activated protein kinase (MAPK) pathway and the nuclear factor *κ*B (NF-*κ*B) pathway signalling cascades [144]. Similarly, NOD receptors also activate the MAPK pathway and NF-*κ*B pathway signalling cascades. Activation and translocation of NF-*κ*B result in the transcription of numerous genes that regulate inflammatory responses. Genes regulated by NF-*κ*B include those encoding cytokines such as interleukins (ILs) and tumour necrosis factors (TNFs). These changes in cytokine production can result in dendritic cell (DC) maturation and activation, which in turn modulates the activation and differentiation of T cells [145, 146]. The specific interactions of MAMPs with PRRs and the subsequent induction of signalling pathways depend on the microorganism and the reactivity of the host, which together play major roles in maintaining the functionality and homeostasis of the intestinal epithelial barrier.

Lactobacilli cell wall components such as LTA and lipoproteins are recognised by TLR2 in combination with TLR6, leading to activation of NF-*κ*B. The two lipid chains of LTA have to be exposed to mediate the interaction with the lipid-binding pocket of TLR2 implying that LTAs may not be key PRR ligands for intestinal epithelial cells [147]. WTA and LTA also bind to macrophage scavenger receptors such as SRA, a type I macrophage scavenger receptor that recognises LTAs, thus contributing to immune signalling [148]. LTA and S-layer protein A (SlpA) interact with DC-specific intercellular adhesion molecule-grabbing nonintegrin (DC-SIGN) on DC to induce cytokine release and T-cell maturation. Activation of DC-SIGN by some strains of lactobacilli affects maturation of DCs, which reduces their capacity to induce IL-10-producing regulatory T-cell responses against pathogens [149]. Glycosylation of SlpA might be necessary for its interaction with DC-SIGN but needs to be validated as DC-SIGN is known to interact with glycosylated ligands of pathogens influencing host response to microorganisms [150]. EPS and other cell wall polysaccharides can be recognised by C-type lectin receptors (CLR) that are present on macrophages and DC. In *L. casei* Shirota, suppression of pro-inflammatory responses in macrophages is mediated by EPS thus indicating an immune suppressive role of cell wall polysaccharides [119]. The ability of lactobacilli to induce host cytokine responses in immune cells can be strikingly different depending on both species and strain. Studies of DC responses to 42 *L. plantarum *strains indicate that cytokines produced can vary from strain to strain, and different strains of the same species can have distinct pro-inflammatory and anti-inflammatory profiles, suggesting that multiple factors can influence immune phenotype [151]. Studies with *L. ruminis* show that some species of lactobacilli display flagella which act as MAMPs that are recognised by the TLR5 of the host and are suggested to activate the NF-*κ*B pathway signalling in epithelial and immune cells of the host [11].

## 5. Strain Specificity and Cell Surface Factors

An understanding of the roles played by bacterial MAMPs (LTA, WTA, PG, and EPS) in strain-specific effects observed in lactobacilli is still developing. Although MAMPs have a similar basic structure in conserved classes of bacterial macromolecules, different microorganisms can display subtle structural variations between MAMPs located on their cell walls. These variations can mean that a macromolecule from one species or strain can act as an agonist for a PRR, whereas a similar macromolecule from another species or strain acts as an antagonist for the same PRR [152]. Studies indicate that adherence characteristics (a major factor in the colonising potential of commensal bacteria) are influenced by cell wall structure and show pronounced variation among strains [9]. Strain specificity is undoubtedly linked to the variability and biochemical complexity of lactobacilli ligands and MAMPs as seen in the substitution levels of TAs, the variable backbone alditol compositions of the WTA, and the modifications of the PG of the cell wall [153]. These modifications in the structure of PG can affect the physiology of the bacterial cell wall by increased sensitivity to autolysis, resistance to lysozyme, and hydrophobicity of the cell envelope which in turn affects recognition by host receptors and bacterial adhesion [33, 34]. 

For example, *L. salivarius* str. Ls33 protects against chemically induced colitis in mice through the interaction of muramyl dipeptides present in its PG with NLR of the intestinal epithelial cells. However, this protective effect is not observed for *L. acidophilus* str. NCFM, as variation in the PG composition of this strain blocks the nucleotide binding domain and leucine-rich repeat containing family (NLR) signalling pathway, which activates the MAPK and NF-*κ*B pathways thereby hindering the activation of host defence mechanisms [154]. Another example of strain-specific characteristics imparted by variation in PG composition is observed in several lactobacilli, where resistance to vancomycin (a glycopeptides antibiotic) was shown to be the result of a replacement of the C-terminal D-alanine residue of MurNAc-pentapeptide by D-lactate [155]. This illustrates the importance of the variable biochemistry of MAMPs such as PG to strain or species specificity. In addition, milieu-dependent switching between the multiple variants of cell wall polymers and/or TAs adds to strain variation in lactobacilli. Studies with mutants of *Lactobacillus* strains that produce alternative LTA variants suggest that modifications to the LTA backbone can alter cytokine induction capacity thus increasing anti-inflammatory immune modulation [156, 157]. Studies with dairy-isolated strains of *L. delbrueckii* showed anti-inflammatory effects *in vitro*, but the extent of these effects varied between strains [158]. These effects are suggested to be linked to the bacterial surface exposed proteins. An interesting observation is that *L. delbrueckii* subspecies bulgaricus 1489 shows poor adherence to Caco-2 epithelial cells implying lack of surface factors in this strain that may be involved in adherence [159]. The high diversity of cell surface components found in lactobacilli adds to strain variation and is reflected in the ecological versatility observed in lactobacilli. Chain length variation, subcellular localisation, and interactions of these polymers most likely contribute to strain-specific characteristics and are still being validated experimentally [50, 160].

## 6. Conclusion

The cell wall is a dynamic entity and plays an essential role in many aspects of the physiology and functioning of lactobacilli. It is where interaction with the bacterial environment occurs, which influences communication and adaptation to host-derived factors encountered in the GIT. Environmental stressors have been shown to affect the cell surface architecture by influencing PG biosynthesis, expression of EPS and cell surface proteins, and LTA decoration with D-alanine residues. Lactobacilli display considerable variation in their cell surface properties, through adaptations which undoubtedly are important for the functioning and survival of these bacteria in the GIT. The increasing possibilities of genomics-based approaches and mutant analyses have resulted in the identification of several effector molecules of lactobacilli. These effector molecules are proposed to be involved in direct interactions with host epithelial or immune cells and many of these effector molecules are components of the cell wall itself [35]. Considering the complexity of host-lactobacilli interactions involving host-cell signalling and regulation pathways, it seems unlikely that single-effector molecules regulate the entire host response. These molecules probably have an expanded repertoire in addition to playing crucial roles as building blocks of the bacterial cell wall [156]. Knowledge of the molecular mechanisms underlying the physiological characteristics of lactobacilli, and identification and validation of effector molecules complemented with parallel studies for their corresponding receptors in the host cells, can strengthen the concept of strain specificity and contributes to the development of strains with enhanced health benefits.

## Figures and Tables

**Table 1:** Lactobacilli cell surface factors implicated in microbe-host interactions.

Lactobacillus strain	Mechanism and effect	Cell surface factors	Target cells or host factors	Reference
*L. reuteri *	Adherence, pathogen inhibition, and enhancement of epithelial barrier function	Mucus binding protein (Mub), collagen binding proteins (CnBP)	Epithelial cells and mucus, collagen	[84, 99, 100]
*L. acidophilus *	Adherence and aggregation, pathogen inhibition, maintenance of barrier function, and immunomodulation	Mucus binding protein (Mub), fibronectin binding protein (FbpA), S-layer proteins (SlpA), LTA, and EPS	Epithelial cells and mucus, fibronectin, ECM components, and Caco-2 cells	[17, 62, 101, 102]
*L. plantarum *	Adherence, enhancement of epithelial barrier function, and immunomodulation	Mannose-specific adhesin (Msa), GAPDH	Epithelial cells and mucus, Caco-2 cells	[103–106]
*L. rhamnosus *	Adherence, protection against pathogen, and antiapoptotic effects on intestinal epithelial cells	Fimbriae, mucus binding factor (MBF)	Mucus glycoproteins, intestinal epithelial cells	[53, 99, 107, 108]
*L. salivarius *	Adherence	Sortase-dependent protein (LspA)	Intestinal epithelial cells and mucus	[109]
*L. crispatus *	Adherence, pathogen inhibition, and resistance to acid and bile	S-layer proteins	HeLa cells	[63]
*L. brevis *	Adherence, protection against stressors (low pH, bile, etc.), and enhancement of barrier function	S-layer proteins (SlpA)	Intestinal epithelial cells	[110, 111]
*L. kefir *	Aggregation and protection against pathogens and stressors	S-layer proteins	Caco-2/TC-7 cells	[112, 113]
*L. fermentum *	Adherence	Mucus binding protein (32-mMubp)	mucus	[114]
*L. johnsonii *	Adherence	LTA, elongation factor Tu (EF-Tu), and heat shock protein (GroEL)	Caco-2 cells, intestinal epithelial cells, and mucus	[102, 115, 116]
*L. ruminis *	Motility, immunomodulation	Flagellin	Intestinal epithelial cells, HT 29, and Caco-2 cells	[11]
*L. casei *	Maintenance of barrier function, increased mucus production, and immunomodulation	EPS, sortase-dependent proteins (SrtA)	Caco-2 cells, HT29 macrophages	[117–119]

## References

[1] Ng SC, Hart AL, Kamm MA, Stagg AJ, Knight SC (2009). Mechanisms of action of probiotics: recent advances. *Inflammatory Bowel Diseases*.

[2] Dethlefsen L, McFall-Ngai M, Relman DA (2007). An ecological and evolutionary perspective on humang-microbe mutualism and disease. *Nature*.

[3] Lebeer S, Vanderleyden J, De Keersmaecker SCJ (2008). Genes and molecules of lactobacilli supporting probiotic action. *Microbiology and Molecular Biology Reviews*.

[4] Sanders TAB (1999). Food production and food safety. *British Medical Journal*.

[5] Remus DM, Kleerebezem M, Bron PA (2011). An intimate tête-à-tête-how probiotic lactobacilli communicate with the host. *European Journal of Pharmacology*.

[6] Ahmed Z, Wang Y, Cheng Q, Imran M (2010). *Lactobacillus* acidophilus bacteriocin, from production to their application: an overview. *African Journal of Biotechnology*.

[7] Hirano J, Yoshida T, Sugiyama T, Koide N, Mori I, Yokochi T (2003). The effect of *Lactobacillus* rhamnosus on enterohemorrhagic Escherichia coli infection of human intestinal cells in vitro. *Microbiology and Immunology*.

[8] Henderson AJ, Kumar A, Barnett B, Dow SW, Ryan EP (2012). Consumption of rice bran increases mucosal immunoglobulin a concentrations and numbers of intestinal *Lactobacillus* spp. *Journal of Medicinal Food*.

[9] Jacobsen CN, Nielsen VR, Hayford AE (1999). Screening of probiotic activities of forty-seven strains of *Lactobacillus* spp. by in vitro techniques and evaluation of the colonization ability of five selected strains in humans. *Applied and Environmental Microbiology*.

[10] Bongaerts GPA, Severijnen RSVM (2001). The beneficial, antimicrobial effect of probiotics. *Medical Hypotheses*.

[11] Neville BA, Forde BM, Claesson MJ (2012). Characterization of pro-inflammatory flagellin proteins produced by *Lactobacillus* ruminis and related motile lactobacilli. *PLoS ONE*.

[12] Maukonen J, Mättö J, Suihko ML, Saarela M (2008). Intra-individual diversity and similarity of salivary and faecal microbiota. *Journal of Medical Microbiology*.

[13] Buck BL, Azcarate-Peril MA, Klaenhammer TR (2009). Role of autoinducer-2 on the adhesion ability of *Lactobacillus* acidophilus. *Journal of Applied Microbiology*.

[14] Servin AL (2004). Antagonistic activities of lactobacilli and bifidobacteria against microbial pathogens. *FEMS Microbiology Reviews*.

[15] Kravtsov EG, Yermolayev AV, Anokhina IV, Yashina NV, Chesnokova VL, Dalin MV (2008). Adhesion characteristics of *Lactobacillus* is a criterion of the probiotic choice. *Bulletin of Experimental Biology and Medicine*.

[16] Vélez MP, De Keersmaecker SCJ, Vanderleyden J (2007). Adherence factors of *Lactobacillus* in the human gastrointestinal tract. *FEMS Microbiology Letters*.

[17] Buck BL, Altermann E, Svingerud T, Klaenhammer TR (2005). Functional analysis of putative adhesion factors in *Lactobacillus* acidophilus NCFM. *Applied and Environmental Microbiology*.

[18] Granato D, Perotti F, Masserey I (1999). Cell surface-associated lipoteichoic acid acts as an adhesion factor for attachment of *Lactobacillus* johnsonii La1 to human enterocyte-like Caco-2 cells. *Applied and Environmental Microbiology*.

[19] Taranto MP, Fernandez Murga ML, Lorca G, De Valdez GF (2003). Bile salts and cholesterol induce changes in the lipid cell membrane of *Lactobacillus* reuteri. *Journal of Applied Microbiology*.

[20] Fozo EM, Kajfasz JK, Quivey RG (2004). Low pH-induced membrane fatty acid alterations in oral bacteria. *FEMS Microbiology Letters*.

[21] Guerzoni ME, Lanciotti R, Cocconcelli PS (2001). Alteration in cellular fatty acid composition as a response to salt, acid, oxidative and thermal stresses in *Lactobacillus* helveticus. *Microbiology*.

[22] Tsai CC, Hsih HY, Chiu HH (2005). Antagonistic activity against Salmonella infection in vitro and in vivo for two *Lactobacillus* strains from swine and poultry. *International Journal of Food Microbiology*.

[23] Forde BM, Neville BA, O'Donnell MM (2011). Genome sequences and comparative genomics of two *Lactobacillus* ruminis strains from the bovine and human intestinal tracts. *Microbial Cell Factories*.

[24] Delcour J, Ferain T, Deghorain M, Palumbo E, Hols P (1999). The biosynthesis and functionality of the cell-wall of lactic acid bacteria. *Antonie van Leeuwenhoek*.

[25] Sánchez B, Schmitter JM, Urdaci MC (2009). Identification of novel proteins secreted by *Lactobacillus* rhamnosus GG grown in de Mann-Rogosa-Sharpe broth. *Letters in Applied Microbiology*.

[26] Sánchez B, Schmitter JM, Urdaci MC (2009). Identification of novel proteins secreted by *Lactobacillus* plantarum That bind to mucin and fibronectin. *Journal of Molecular Microbiology and Biotechnology*.

[27] Dijkstra AJ, Keck W (1996). Peptidoglycan as a barrier to transenvelope transport. *Journal of Bacteriology*.

[28] Asong J, Wolfert MA, Maiti KK, Miller D, Boons GJ (2009). Binding and cellular activation studies reveal that toll-like receptor 2 can differentially recognize peptidoglycan from gram-positive and gram-negative bacteria. *Journal of Biological Chemistry*.

[29] Veiga P, Piquet S, Maisons A (2006). Identification of an essential gene responsible for D-Asp incorporation in the Lactococcus lactis peptidoglycan crossbridge. *Molecular Microbiology*.

[30] Araki Y, Oba S, Ito E, Araki S (1980). Enzymatic deacetylation of N-acetylglucosamine residues in cell wall peptidoglycan. *Journal of Biochemistry*.

[31] Clarke AJ, Dupont C (1992). O-Acetylated peptidoglycan: its occurrence, pathobiological significance, and biosynthesis. *Canadian Journal of Microbiology*.

[32] Archibald AR (1985). Structure and assembly of the cell wall in Bacillus subtilis. *Biochemical Society Transactions*.

[33] Hamada S, Torii M, Kotani S (1978). Lysis of Streptococcus mutans cells with mutanolysin, a lytic enzyme prepared from a culture liquor of Streptomyces globisporus 1829. *Archives of Oral Biology*.

[34] Rosenberg M, Kjelleberg S (1986). Hydrophobic interactions: role in bacterial adhesion. *Microbiology Ecology*.

[35] Kleerebezem M, Hols P, Bernard E (2010). The extracellular biology of the lactobacilli. *FEMS Microbiology Reviews*.

[36] Bron PA, Tomita S, van Swam II (2012). *Lactobacillus* plantarum possesses the capability for wall teichoic acid backbone alditol switching. *Microbial Cell Factories*.

[37] Tomita S, Irisawa T, Tanaka N (2010). Comparison of components and synthesis genes of cell wall teichoic acid among *Lactobacillus* plantarum strains. *Bioscience, Biotechnology and Biochemistry*.

[38] Andre G, Deghorain M, Bron PA (2011). Fluorescence and atomic force microscopy imaging of wall teichoic acids in *Lactobacillus* plantarum. *ACS Chemical Biology*.

[39] Vélez MP, Verhoeven TLA, Draing C (2007). Functional analysis of D-alanylation of lipoteichoic acid in the probiotic strain *Lactobacillus* rhamnosus GG. *Applied and Environmental Microbiology*.

[40] Grant WD (1979). Cell wall teichoic acid as a reserve phosphate source in Bacillus subtilis. *Journal of Bacteriology*.

[41] Hughes AH, Hancock IC, Baddiley J (1973). The function of teichoic acids in cation control in bacterial membranes. *Biochemical Journal*.

[42] Holtje JV, Tomasz A (1975). Lipoteichoic acid: a specific inhibitor of autolysin activity in pneumococcus. *Proceedings of the National Academy of Sciences of the United States of America*.

[43] Räisänen L, Schubert K, Jaakonsaari T, Alatossava T (2004). Characterization of lipoteichoic acids as *Lactobacillus* delbrueckii phage receptor components. *Journal of Bacteriology*.

[44] Gopal PK, Crow VL (1993). Characterization of loosely associated material from the cell surface of Lactococcus lactis subsp. cremoris E8 and its phage-resistant variant strain 398. *Applied and Environmental Microbiology*.

[45] Whitfield C (1988). Bacterial extracellular polysaccharides. *Canadian Journal of Microbiology*.

[46] Reeves PR, Hobbs M, Valvano MA (1996). Bacterial polysaccharide synthesis and gene nomenclature. *Trends in Microbiology*.

[47] Wicken AJ, Ayres A, Campbell LK, Knox KW (1983). Effect of growth conditions on production of rhamnose-containing cell wall and capsular polysaccharides by strains of *Lactobacillus* casei subsp. rhamnosus. *Journal of Bacteriology*.

[48] De Vuyst L, De Vin F, Vaningelgem F, Degeest B (2001). Recent developments in the biosynthesis and applications of heteropolysaccharides from lactic acid bacteria. *International Dairy Journal*.

[49] Tieking M, Kaditzky S, Valcheva R, Korakli M, Vogel RF, Gänzle MG (2005). Extracellular homopolysaccharides and oligosaccharides from intestinal lactobacilli. *Journal of Applied Microbiology*.

[50] Francius G, Lebeer S, Alsteens D (2008). Detection, localization, and conformational analysis of single polysaccharide molecules on live bacteria. *ACS Nano*.

[51] Ciszek-Lenda M, Strus M, Górska-Frączek S (2011). Strain specific immunostimulatory potential of lactobacilli-derived exopolysaccharides. *Central-European Journal of Immunology*.

[52] Lebeer S, Claes IJJ, Verhoeven TLA, Vanderleyden J, De Keersmaecker SCJ (2011). Exopolysaccharides of *Lactobacillus* rhamnosus GG form a protective shield against innate immune factors in the intestine. *Microbial Biotechnology*.

[53] Kankainen M, Paulin L, Tynkkynen S (2009). Comparative genomic analysis of *Lactobacillus* rhamnosus GG reveals pili containing a human-mucus binding protein. *Proceedings of the National Academy of Sciences of the United States of America*.

[54] Reunanen J, von Ossowski I, Hendrickx APA, Palva A, de Vosa WM (2012). Characterization of the SpaCBA pilus fibers in the probiotic *Lactobacillus* rhamnosus GG. *Applied and Environmental Microbiology*.

[55] Mandlik A, Swierczynski A, Das A, Ton-That H (2008). Pili in Gram-positive bacteria: assembly, involvement in colonization and biofilm development. *Trends in Microbiology*.

[56] Budzik JM, Oh SY, Schneewind O (2009). Sortase D forms the covalent bond that links BcpB to the tip of Bacillus cereus pili. *Journal of Biological Chemistry*.

[57] Scott JR, Zähner D (2006). Pili with strong attachments: gram-positive bacteria do it differently. *Molecular Microbiology*.

[58] Lebeer S, Claes I, Tytgat HLP (2012). Functional analysis of *Lactobacillus* rhamnosus GG pili in relation to adhesion and immunomodulatory interactions with intestinal epithelial cells. *Applied and Environmental Microbiology*.

[59] Danne C, Dramsi S (2012). Pili of Gram-positive bacteria: roles in host colonization. *Research in Microbiology*.

[60] Tallant T, Deb A, Kar N, Lupica J, De Veer MJ, DiDonato JA (2004). Flagellin acting via TLR5 is the major activator of key signaling pathways leading to NF-*κ*B and proinflammatory gene program activation in intestinal epithelial cells. *BMC Microbiology*.

[61] Båth K, Roos S, Wall T, Jonsson H (2005). The cell surface of *Lactobacillus* reuteri ATCC 55730 highlighted by identification of 126 extracellular proteins from the genome sequence. *FEMS Microbiology Letters*.

[62] Lorca G, Torino MI, Font de Valdez G, Ljungh A (2002). Lactobacilli express cell surface proteins which mediate binding of immobilized collagen and fibronectin. *FEMS Microbiology Letters*.

[63] Chen X, Xu J, Shuai J, Chen J, Zhang Z, Fang W (2007). The S-layer proteins of *Lactobacillus* crispatus strain ZJ001 is responsible for competitive exclusion against Escherichia coli O157:H7 and Salmonella typhimurium. *International Journal of Food Microbiology*.

[64] Wells JM (2011). Immunomodulatory mechanisms of lactobacilli. *Microbial Cell Factories*.

[65] Hutchings MI, Palmer T, Harrington DJ, Sutcliffe IC (2009). Lipoprotein biogenesis in Gram-positive bacteria: knowing when to hold ’em, knowing when to fold ’em. *Trends in Microbiology*.

[66] Marraffini LA, Dedent AC, Schneewind O (2006). Sortases and the art of anchoring proteins to the envelopes of gram-positive bacteria. *Microbiology and Molecular Biology Reviews*.

[67] Navarre WW, Schneewind O (1994). Proteolytic cleavage and cell wall anchoring at the LPXTG motif of surface proteins in Gram-positive bacteria. *Molecular Microbiology*.

[68] Mazmanian SK, Ton-That H, Su K, Schneewind O (2002). An iron-regulated sortase anchors a class of surface protein during Staphylococcus aureus pathogenesis. *Proceedings of the National Academy of Sciences of the United States of America*.

[69] Comfort D, Clubb RT (2004). A comparative genome analysis identifies distinct sorting pathways in Gram-positive bacteria. *Infection and Immunity*.

[70] Åvall-Jääskeläinen S, Palva A (2005). *Lactobacillus* surface layers and their applications. *FEMS Microbiology Reviews*.

[71] Mobili P, de los Ángeles Serradell M, Trejo SA, Avilés Puigvert FX, Abraham AG, De Antoni GL (2009). Heterogeneity of S-layer proteins from aggregating and non-aggregating *Lactobacillus* kefir strains. *Antonie van Leeuwenhoek*.

[72] Lortal S, Van Heijenoort J, Gruber K, Sleytr UB (1992). S-layer of *Lactobacillus* helveticus ATCC 12046: isolation, chemical characterization and re-formation after extraction with lithium chloride. *Journal of General Microbiology*.

[73] Wren BW (1991). A family of clostridial and streptococcal ligand-binding proteins with conserved C-terminal repeat sequences. *Molecular Microbiology*.

[74] Hidalgo IJ, Raub TJ, Borchardt RT (1989). Characterization of the human colon carcinoma cell line (Caco-2) as a model system for intestinal epithelial permeability. *Gastroenterology*.

[75] Boekhorst J, Wels M, Kleeberezem M, Siezen RJ (2006). The predicted secretome of *Lactobacillus* plantarum WCFS1 sheds light on interactions with its environment. *Microbiology*.

[76] Brinster S, Furlan S, Serror P (2007). C-terminal WxL domain mediates cell wall binding in Enterococcus faecalis and other gram-positive bacteria. *Journal of Bacteriology*.

[77] Lu JZ, Fujiwara T, Komatsuzawa H, Sugai M, Sakon J (2006). Cell wall-targeting domain of glycylglycine endopeptidase distinguishes among peptidoglycan cross-bridges. *Journal of Biological Chemistry*.

[78] Lorca GL, Raya RR, Taranto MP, De Valdez GF (1998). Adaptive acid tolerance response in *Lactobacillus* acidophilus. *Biotechnology Letters*.

[79] De Smet I, Van Hoorde L, Vande Woestyne M, Christiaens H, Verstraete W (1995). Significance of bile salt hydrolytic activities of lactobacilli. *Journal of Applied Bacteriology*.

[80] Moser SA, Savage DC (2001). Bile salt hydrolase activity and resistance to toxicity of conjugated bile salts are unrelated properties in Lactobacilli. *Applied and Environmental Microbiology*.

[81] Begley M, Gahan CGM, Hill C (2005). The interaction between bacteria and bile. *FEMS Microbiology Reviews*.

[82] Pfeiler EA, Azcarate-Peril MA, Klaenhammer TR (2007). Characterization of a novel bile-inducible operon encoding a two-component regulatory system in *Lactobacillus* acidophilus. *Journal of Bacteriology*.

[83] Wall T, Båth K, Britton RA, Jonsson H, Versalovic J, Roos S (2007). The early response to acid shock in *Lactobacillus* reuteri involves the ClpL chaperone and a putative cell wall-altering esterase. *Applied and Environmental Microbiology*.

[84] Whitehead K, Versalovic J, Roos S, Britton RA (2008). Genomic and genetic characterization of the bile stress response of probiotic *Lactobacillus* reuteri ATCC 55730. *Applied and Environmental Microbiology*.

[85] Neuhaus FC, Baddiley J (2003). A continuum of anionic charge: structures and functions of d-alanyl-teichoic acids in Gram-positive bacteria. *Microbiology and Molecular Biology Reviews*.

[86] Miyoshi A, Rochat T, Gratadoux JJ (2003). Oxidative stress in Lactococcus lactis. *Genetics and Molecular Research*.

[87] van de Guchte M, Serror P, Chervaux C, Smokvina T, Ehrlich SD, Maguin E (2002). Stress responses in lactic acid bacteria. *Antonie van Leeuwenhoek*.

[88] Masip L, Veeravalli K, Georgiou G (2006). The many faces of glutathione in bacteria. *Antioxidants and Redox Signaling*.

[89] Altermann E, Buck LB, Cano R, Klaenhammer TR (2004). Identification and phenotypic characterization of the cell-division protein CdpA. *Gene*.

[90] Klaenhammer TR, Barrangou R, Buck BL, Azcarate-Peril MA, Altermann E (2005). Genomic features of lactic acid bacteria effecting bioprocessing and health. *FEMS Microbiology Reviews*.

[91] Zink R, Walker C, Schmidt G, Elli M, Pridmore D, Reniero R (2000). Impact of multiple stress factors on the survival of dairy lactobacilli. *Sciences des Aliments*.

[92] Chatterji D, Kumar Ojha A (2001). Revisiting the stringent response, ppGpp and starvation signaling. *Current Opinion in Microbiology*.

[93] Konings WN, Lolkema JS, Bolhuis H, Van Veen HW, Poolman B, Driessen AJM (1997). The role of transport processes in survival of lactic acid bacteria. Energy transduction and multidrug resistance. *Antonie van Leeuwenhoek*.

[94] Champomier-Vergès MC, Chaillou S, Cornet M, Zagorec M (2001). *Lactobacillus* sakei: recent developments and future prospects. *Research in Microbiology*.

[95] Lorca GL, Font De Valdez G (2001). Acid tolerance mediated by membrane ATPases in *Lactobacillus* acidophilus. *Biotechnology Letters*.

[96] Hartke A, Bouche S, Gansel X, Boutibonnes P, Auffray Y (1994). Starvation-induced stress resistance in Lactococcus lactis subsp. lactis IL1403. *Applied and Environmental Microbiology*.

[97] Chervaux C, Ehrlich SD, Maguin E (2000). Physiological study of *Lactobacillus* delbrueckii subsp. bulgaricus strains in a novel chemically defined medium. *Applied and Environmental Microbiology*.

[98] Gallo RL, Hooper LV (2012). Epithelial antimicrobial defence of the skin and intestine. *Nature Reviews Immunology*.

[99] Lebeer S, Verhoeven TLA, Francius G (2009). Identification of a gene cluster for the biosynthesis of a long, galactose-rich exopolysaccharide in *Lactobacillus* rhamnosus GG and functional analysis of the priming glycosyltransferase. *Applied and Environmental Microbiology*.

[100] Schneitz C, Nuotio L, Lounatmaa K (1993). Adhesion of *Lactobacillus* acidophilus to avian intestinal epithelial cells mediated by the crystalline bacterial cell surface layer (S-layer). *Journal of Applied Bacteriology*.

[101] Coconnier MH, Klaenhammer TR, Kerneis S, Bernet MF, Servin AL (1992). Protein-mediated adhesion of *Lactobacillus* acidophilus BG2FO4 on human enterocyte and mucus-secreting cell lines in culture. *Applied and Environmental Microbiology*.

[102] Vidal K, Donnet-Hughes A, Granato D (2002). Lipoteichoic acids from *Lactobacillus* johnsonii strain La1 and *Lactobacillus* acidophilus strain La10 antagonize the responsiveness of human intestinal epithelial HT29 cells to lipopolysaccharide and gram-negative bacteria. *Infection and Immunity*.

[103] Pretzer G, Snel J, Molenaar D (2005). Biodiversity-based identification and functional characterization of the mannose-specific adhesin of *Lactobacillus* plantarum. *Journal of Bacteriology*.

[104] Kinoshita H, Uchida H, Kawai Y (2008). Cell surface *Lactobacillus* plantarum LA 318 glyceraldehyde-3-phosphate dehydrogenase (GAPDH) adheres to human colonic mucin. *Journal of Applied Microbiology*.

[105] Mao Y, Nobaek S, Kasravi B (1996). The effects of *Lactobacillus* strains and oat fiber on methotrexate- induced enterocolitis in rats. *Gastroenterology*.

[106] Anderson RC, Cookson AL, McNabb WC, Kelly WJ, Roy NC (2010). *Lactobacillus* plantarum DSM 2648 is a potential probiotic that enhances intestinal barrier function. *FEMS Microbiology Letters*.

[107] von Ossowski I, Satokari R, Reunanen J (2011). Functional characterization of a mucus-specific LPXTG surface adhesin from probiotic *Lactobacillus* rhamnosus GG. *Applied and Environmental Microbiology*.

[108] Yan F, Polk DB (2002). Probiotic bacterium prevents cytokine-induced apoptosis in intestinal epithelial cells. *Journal of Biological Chemistry*.

[109] van Pijkeren JP, Canchaya C, Ryan KA (2006). Comparative and functional analysis of sortase-dependent proteins in the predicted secretome of *Lactobacillus* salivarius UCC118. *Applied and Environmental Microbiology*.

[110] Åvall-Jääskeläinen S, Lindholm A, Palva A (2003). Surface display of the receptor-binding region of the *Lactobacillus* brevis S-layer protein in Lactococcus lactis provides nonadhesive lactococci with the ability to adhere to intestinal epithelial cells. *Applied and Environmental Microbiology*.

[111] Garcia-Lafuente A, Antolin M, Guarner F, Crespo E, Malagelada JR (2001). Modulation of colonic barrier function by the composition of the commensal flora in the rat. *Gut*.

[112] Golowczyc MA, Mobili P, Garrote GL, Abraham AG, De Antoni GL (2007). Protective action of *Lactobacillus* kefir carrying S-layer protein against Salmonella enterica serovar Enteritidis. *International Journal of Food Microbiology*.

[113] Golowczyc MA, Mobili P, Garrote GL, De Los Angeles Serradell M, Abraham AG, De Antoni GL (2009). Interaction between *Lactobacillus* kefir and saccharomyces lipolytica isolated from kefir grains: evidence for lectin-like activity of bacterial surface proteins. *Journal of Dairy Research*.

[114] MacÍas-Rodríguez ME, Zagorec M, Ascencio F, Vázquez-Juárez R, Rojas M (2009). *Lactobacillus* fermentum BCS87 expresses mucus- and mucin-binding proteins on the cell surface. *Journal of Applied Microbiology*.

[115] Granato D, Bergonzelli GE, Pridmore RD, Marvin L, Rouvet M, Corthésy-Theulaz IE (2004). Cell surface-associated elongation factor Tu mediates the attachment of *Lactobacillus* johnsonii NCC533 (La1) to human intestinal cells and mucins. *Infection and Immunity*.

[116] Bergonzelli GE, Granato D, Pridmore RD, Marvin-Guy LF, Donnicola D, Corthésy-Theulaz IE (2006). GroEL of *Lactobacillus* johnsonii La1 (NCC 533) is cell surface associated: potential role in interactions with the host and the gastric pathogen Helicobacter pylori. *Infection and Immunity*.

[117] Muñoz-Provencio D, Rodríguez-Díaz J, Collado MC (2012). Functional analysis of the *Lactobacillus* casei BL23 sortases. *Applied and Environmental Microbiology*.

[118] Mattar AF, Teitelbaum DH, Drongowski RA, Yongyi F, Harmon CM, Coran AG (2002). Probiotics up-regulate MUC-2 mucin gene expression in a Caco-2 cell-culture model. *Pediatric Surgery International*.

[119] Yasuda E, Serata M, Sako T (2009). Suppressive effect on activation of macrophages by *Lactobacillus* casei strain shirota genes determining the synthesis of cell wall-associated polysaccharides (Applied and Environmental Microbiology (2008) 74, 15, (4746-4755)). *Applied and Environmental Microbiology*.

[120] Garmasheva IL, Kovalenko NK (2005). Adhesive properties of lactic acid bacteria and methods of their investigation. *Mikrobiolohichnyi Zhurnal*.

[121] Gueimonde M, Jalonen L, He F, Hiramatsu M, Salminen S (2006). Adhesion and competitive inhibition and displacement of human enteropathogens by selected lactobacilli. *Food Research International*.

[122] MacKenzie DA, Jeffers F, Parker ML (2010). Strain-specific diversity of mucus-binding proteins in the adhesion and aggregation properties of *Lactobacillus* reuteri. *Microbiology*.

[123] Tuomola EM, Ouwehand AC, Salminen SJ (2000). Chemical, physical and enzymatic pre-treatments of probiotic lactobacilli alter their adhesion to human intestinal mucus glycoproteins. *International Journal of Food Microbiology*.

[124] Macfarlane S, Dillon JF (2007). Microbial biofilms in the human gastrointestinal tract. *Journal of Applied Microbiology*.

[125] Roos S, Jonsson H (2002). A high-molecular-mass cell-surface protein from *Lactobacillus* reuteri 1063 adheres to mucus components. *Microbiology*.

[126] Boekhorst J, Helmer Q, Kleerebezem M, Siezen RJ (2006). Comparative analysis of proteins with a mucus-binding domain found exclusively in lactic acid bacteria. *Microbiology*.

[127] Barocchi MA, Ries J, Zogaj X (2006). A pneumococcal pilus influences virulence and host inflammatory responses. *Proceedings of the National Academy of Sciences of the United States of America*.

[128] Canchaya C, Claesson MJ, Fitzgerald GF, van Sinderen D, O’Toole PW (2006). Diversity of the genus *Lactobacillus* revealed by comparative genomics of five species. *Microbiology*.

[129] Kos B, Šušković J, Vuković S, Šimpraga M, Frece J, Matošić S (2003). Adhesion and aggregation ability of probiotic strain *Lactobacillus* acidophilus M92. *Journal of Applied Microbiology*.

[130] Aleljung P, Shen W, Rozalska B, Hellman U, Ljungh A, Wadstrom T (1994). Purification of collagen-binding proteins of *Lactobacillus* reuteri NCIB 11951. *Current Microbiology*.

[131] Denou E, Pridmore RD, Berger B, Panoff JM, Arigoni F, Brüssow H (2008). Identification of genes associated with the long-gut-persistence phenotype of the probiotic *Lactobacillus* johnsonii strain NCC533 using a combination of genomics and transcriptome analysis. *Journal of Bacteriology*.

[132] Ruas-Madiedo P, Gueimonde M, Margolles A, De Los Reyes-Gavilán CG, Salminen S (2006). Exopolysaccharides produced by probiotic strains modify the adhesion of probiotics and enteropathogens to human intestinal mucus. *Journal of Food Protection*.

[133] Madsen KL, Doyle JS, Jewell LD, Tavernini MM, Fedorak RN (1999). *Lactobacillus* species prevents colitis in interleukin 10 gene-deficient mice. *Gastroenterology*.

[134] Claes IJJ, Segers ME, Verhoeven TLA (2012). Lipoteichoic acid is an important microbe-associated molecular pattern of *Lactobacillus* rhamnosus GG. *Microbial Cell Factories*.

[135] Mack DR, Ahrne S, Hyde L, Wei S, Hollingsworth MA (2003). Extracellular MUC3 mucin secretion follows adherence of *Lactobacillus* strains to intestinal epithelial cells in vitro. *Gut*.

[136] Mack DR, Michail S, Wei S, McDougall L, Hollingsworth MA (1999). Probiotics inhibit enteropathogenic E. coli adherence in vitro by inducing intestinal mucin gene expression. *American Journal of Physiology*.

[137] Resta-Lenert S, Barrett KE (2003). Live probiotics protect intestinal epithelial cells from the effects of infection with enteroinvasive Escherichia coli (EIEC). *Gut*.

[138] Qin HL, Shen TY, Gao ZG (2005). Effect of *Lactobacillus* on the gut microflora and barrier function of the rats with abdominal infection. *World Journal of Gastroenterology*.

[139] Qin H, Zhang Z, Hang X, Jiang Y (2009). L. plantarum prevents Enteroinvasive Escherichia coli-induced tight junction proteins changes in intestinal epithelial cells. *BMC Microbiology*.

[140] Sherman PM, Johnson-Henry KC, Yeung HP, Ngo PSC, Goulet J, Tompkins TA (2005). Probiotics reduce enterohemorrhagic Escherichia coli O157:H7- and enteropathogenic E. coli O127:H6-induced changes in polarized T84 epithelial cell monolayers by reducing bacterial adhesion and cytoskeletal rearrangements. *Infection and Immunity*.

[141] Rousseaux C, Thuru X, Gelot A (2007). *Lactobacillus* acidophilus modulates intestinal pain and induces opioid and cannabinoid receptors. *Nature Medicine*.

[142] Kawai T, Akira S (2010). The role of pattern-recognition receptors in innate immunity: update on toll-like receptors. *Nature Immunology*.

[143] Artis D (2008). Epithelial-cell recognition of commensal bacteria and maintenance of immune homeostasis in the gut. *Nature Reviews Immunology*.

[144] Janssens S, Beyaert R (2002). A universal role for MyD88 in TLR/IL-1R-mediated signaling. *Trends in Biochemical Sciences*.

[145] De Jong EC, Smits HH, Kapsenberg ML (2005). Dendritic cell-mediated T cell polarization. *Springer Seminars in Immunopathology*.

[146] Rescigno M (2010). Intestinal dendritic cells. *Advances in Immunology C*.

[147] Jin MS, Kim SE, Heo JY (2007). Crystal Structure of the TLR1-TLR2 Heterodimer Induced by Binding of a Tri-Acylated Lipopeptide. *Cell*.

[148] Dunne DW, Resnick D, Greenberg J, Krieger M, Joiner KA (1994). The type I macrophage scavenger receptor binds to Gram-positive bacteria and recognizes lipoteichoic acid. *Proceedings of the National Academy of Sciences of the United States of America*.

[149] Smits HH, Engering A, Van Der Kleij D (2005). Selective probiotic bacteria induce IL-10-producing regulatory T cells in vitro by modulating dendritic cell function through dendritic cell-specific intercellular adhesion molecule 3-grabbing nonintegrin. *Journal of Allergy and Clinical Immunology*.

[150] van Kooyk Y, Geijtenbeek TBH (2003). DC-SIGN: escape mechanism for pathogens. *Nature Reviews Immunology*.

[151] Meijerink M, van Hemert S, Taverne N (2010). Identification of genetic loci in *Lactobacillus* plantarum that modulate the immune response of dendritic cells using comparative genome hybridization. *PLoS ONE*.

[152] Erridge C, Pridmore A, Eley A, Stewart J, Poxton IR (2004). Lipopolysaccharides of Bacteroides fragilis, Chlamydia trachomatis and Pseudomonas aeruginosa signal via Toll-like receptor 2. *Journal of Medical Microbiology*.

[153] Bron PA, Van Baarlen P, Kleerebezem M (2012). Emerging molecular insights into the interaction between probiotics and the host intestinal mucosa. *Nature Reviews Microbiology*.

[154] Macho Fernandez E, Valenti V, Rockel C (2011). Anti-inflammatory capacity of selected lactobacilli in experimental colitis is driven byNOD2-mediated recognition of a specific peptidoglycan-derived muropeptide (Gut (2011) 60, (1050–1059)). *Gut*.

[155] Arthur M, Molinas C, Bugg TDH, Wright GD, Walsh CT, Courvalin P (1992). Evidence for in vivo incorporation of D-lactate into peptidoglycan precursors of vancomycin-resistant enterococci. *Antimicrobial Agents and Chemotherapy*.

[156] Grangette C, Nutten S, Palumbo E (2005). Enhanced antiinflammatory capacity of a *Lactobacillus* plantarum mutant synthesizing modified teichoic acids. *Proceedings of the National Academy of Sciences of the United States of America*.

[157] Saber R, Zadeh M, Pakanati KC, Bere P, Klaenhammer T, Mohamadzadeh M (2011). Lipoteichoic acid-deficient *Lactobacillus* acidophilus regulates downstream signals. *Immunotherapy*.

[158] Rocha CS, Lakhdari O, Blottière HM (2012). Anti-inflammatory properties of dairy lactobacilli. *Inflammatory Bowel Diseases*.

[159] Greene JD, Klaenhammer TR (1994). Factors involved in adherence of lactobacilli to human Caco-2 cells. *Applied and Environmental Microbiology*.

[160] Kaji R, Kiyoshima-Shibata J, Nagaoka M, Nanno M, Shida K (2010). Bacterial teichoic acids reverse predominant IL-12 production induced by certain *Lactobacillus* strains into predominant IL-10 production via TLR2-dependent ERK activation in macrophages. *Journal of Immunology*.

